# Supplementary material for: Mental health is just an Addendum: Assessing stakeholder’s perceptions on COVID-19 and mental health services provision in Malawi
Source: PLoS One. 2024 Jun 13;19(6):e0305341. doi: 10.1371/journal.pone.0305341 (PMC11175459; doi:10.1371/journal.pone.0305341)
Supplement: S1 File — (DOCX) [file pone.0305341.s001.docx]

**
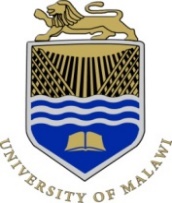
**
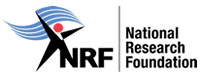
**COVID-19 AND MENTAL HEALTH IN MALAWI**

**Key Informant Interview Guide**

**For Mental Health Policy Makers, Leaders and Managers- Ministry of Health/DHO/Pvt and Public Mental Health Experts**

|  | Name of enumerator |  |
| --- | --- | --- |
|  | Code of Respondent/ QID |  |
|  | Institutional affiliation |  |
|  | District |  |
|  | Date of interview |  |

1. Can you tell us about your knowledge with regard to the availability of mental health services in Malawi?
2. What is your main funding source for the provision of mental health services? And is the funding adequate?
3. Can you tell us the role that your organization/institution is playing with regards to the provision of mental health services and mental health service during Covid-19?
4. Are you aware of any clinical guidelines that guide the provision of mental health services in Malawi? Explain
5. What are the main national laws, policies and strategies as well as assessments related to mental health that you are aware of?
6. Is there a cadre of providers who are currently qualified and permitted to provide mental health services such as counselling, medication prescription at the facility level? At the community level?
7. How has Covid-19 affected the provision/delivery of mental health services in Malawi?
8. What challenges has mental health service provision met in light of Covid-19?
9. What opportunities have been created by Covid-19 on mental health service provision in Malawi?
10. Has Covid-19 revealed any gaps in mental health service delivery in Malawi?
11. What governance structures and systems do you think should be in place to ensure we close the gaps that have been revealed by Covid-19 and for effective provision of mental health services in Malawi?
