# Supplementary material for: Mental health is just an Addendum: Assessing stakeholder’s perceptions on COVID-19 and mental health services provision in Malawi
Source: PLoS One. 2024 Jun 13;19(6):e0305341. doi: 10.1371/journal.pone.0305341 (PMC11175459; doi:10.1371/journal.pone.0305341)
Supplement: Attachment — Submitted filename: Review-Plos1-March-23.doc [file pone.0305341.s002.doc]

This study aimed to assess the stakeholders’ perception on the state of mental health services provision in Malawi and how the COVID-19 pandemic could have impacted the mental health sector. Taking the scarcity of literature on this phenomenon, the authors clearly reviewed the existing literature and brought the rationale for their study into focus. Given the novelty of such studies about the impact of covid-19 on both health systems and individual mental wellbeing, the authors are commended for coming up with such work. I however have a few comments that could improve the manuscript before its published

Abstract:

**Methods**

When I see a statement like “We purposively sampled **nine** stakeholders” it automatically appears to me that the sample was pre-determined and yet, the sample size should have been determined at a saturation point.

**Conclusion:** The authors only state the conclusion from the participants(e.g.,) “The stakeholders also recommended integration of mental health services in all levels of care to improve service quality and efficiency’ Information gathered from the participants should only appear in the result’s section. I wonder why the author’s personal conclusive remarks based on the study’s findings are missing.

**Methodology.**

Although I am cognizant that saturation can be reached by interviewing few participants especially using phenomenological approaches, the authors should provide detailed information in the methods why their sample size was reached at nine participants and yet there are several other significant stake holders that could have participants in this study. Unless if some professions aren’t considered in the country’s mental health systems structure, I miss to see Psychiatrists/Consultants/Psychologists and Psychiatry Clinical officers and more nurses at different facility levels.

**Results.**

I suggest that Table1 be moved to results section. Otherwise, it still appears to me that the sample size was pre-determined. Even though the study is qualitative, it would improve their manuscript, if the authors added more information on the participants demographics e.g., age, time spent in service, qualifications, levels of health facilities and any other relevant information.

**Discussion**

Ok
