# Supplementary material for: Mental health is just an Addendum: Assessing stakeholder’s perceptions on COVID-19 and mental health services provision in Malawi
Source: PLoS One. 2024 Jun 13;19(6):e0305341. doi: 10.1371/journal.pone.0305341 (PMC11175459; doi:10.1371/journal.pone.0305341)
Supplement: Attachment — Submitted filename: Rebuttal Letter.doc [file pone.0305341.s003.doc]

Frank Kyei-Arthur, Ph.D

Academic Editor
PLOS ONE

# 19/05/2023

# Dear Dr. Kyei-Arthur,

# Thank you for inviting us to submit a revised draft of our manuscript entitled, " *Mental Health is Just an Addendum*: Assessing stakeholders’ perceptions on mental health services provision in Malawi prior to and during COVID-19" to PLOS ONE. We also appreciate the time and effort you and each of the reviewers have dedicated to providing insightful feedback on ways to strengthen our paper. Thus, it is with great pleasure that we resubmit our article for further consideration. We have incorporated changes that reflect the detailed suggestions you have graciously provided. We also hope that our edits and the responses we provide below satisfactorily address all the issues and concerns you and the reviewers have noted.

To facilitate your review of our revisions, the following is a point-by-point response to the questions and comments delivered in your letter dated 18 April, 2023.

# A. Editor’s comments

1. The abstract

Response- This has been revised

2. The methods (sampling approach, sample size, and selection of participants for study)

Response- We made clarifications on this and responded to the reviewers’ query

3. The results (enhancing the socio-demographic information of participants, and comparing findings on "prior to" and "during COVID-19"

Response- The title has been revised to reflect the content captured in the study. We have also made clarifications on why for this type of study and audience we did not collect data on demographic characteristics of the respondents.

4. Addressing previous comment on why the study did not covered the effectiveness of mental health services?.

Response- Thank you Editor, this has been addressed.

# B. Reviewer 4

This study aimed to assess the stakeholders’ perception on the state of mental health services provision in Malawi and how the COVID-19 pandemic could have impacted the mental health sector. Taking the scarcity of literature on this phenomenon, the authors clearly reviewed the existing literature and brought the rationale for their study into focus. Given the novelty of such studies about the impact of covid-19 on both health systems and individual mental wellbeing, the authors are commended for coming up with such work. I however have a few comments that could improve the manuscript before its published

Abstract:

1. **Methods.** When I see a statement like “We purposively sampled **nine** stakeholders” it automatically appears to me that the sample was pre-determined and yet, the sample size should have been determined at a saturation point.

**Response-** in this study**,** we utilised purposive sampling to differentiate it from random sampling. We deliberately selected individuals with experience in the mental health sector for the interviews, and the number of participants was determined based on data saturation. The decision to use purposive sampling was solely based on the characteristics of our target population, and not on the number of interviews conducted.

To avoid any confusion, we have rephrased the sentence to provide a clear explanation of the concept of purposive sampling as used in this study. Find the paraphrased sentence below

*“We conducted a formative qualitative assessment using key informant interviews (KIIs) among stakeholders with expert knowledge and involvement of mental health services in Malawi. Data were collected till saturation point”.*

*Also refer to the systematic review* [*here*](https://www.sciencedirect.com/science/article/pii/S0277953621008558) *that found that a sample size of between 9-17 interviews reached saturation*

2. **Conclusion:** The authors only state the conclusion from the participants(e.g.,) “The stakeholders also recommended integration of mental health services in all levels of care to improve service quality and efficiency’ Information gathered from the participants should only appear in the result’s section. I wonder why the author’s personal conclusive remarks based on the study’s findings are missing.

**Response**- Thank you so much for this comment. The conclusion has been revised to reflect our personal conclusive remarks. Find below the rewritten section of the abstract that captures our reflections.

*“To address these challenges, policymakers in Malawi could prioritise mental health and integrate mental health services into primary healthcare services to expand access Additionally, policymakers could explore partnerships with the private sector to address shortages of mental health professionals and medication. Overall, this study highlights the need for policymakers to invest in the mental health sector to improve service quality and efficiency, particularly in light of the COVID-19 pandemic”.*

3. **Methodology.** Although I am cognizant that saturation can be reached by interviewing few participants especially using phenomenological approaches, the authors should provide detailed information in the methods why their sample size was reached at nine participants and yet there are several other significant stake holders that could have participants in this study. Unless if some professions aren’t considered in the country’s mental health systems structure, I miss to see Psychiatrists/Consultants/Psychologists and Psychiatry Clinical officers and more nurses at different facility levels.

**Response**- Thank you for your valuable feedback on our methodology. We appreciate your concern regarding our sample size of nine participants and the exclusion of certain stakeholder groups from our study. We agree that it is important to provide a detailed explanation of our sample size and stakeholder selection process.

In our study, we aimed to explore the state of mental health services in Malawi, with a specific focus on the challenges and opportunities in the sector. As we mentioned in our paper, the mental health system in Malawi is still developing, and there are only a limited number of facilities and stakeholders available for us to draw from. You can see from this reflective [essay](https://www.ncbi.nlm.nih.gov/pmc/articles/PMC7283112/) that the public facilities have three trained psychiatric nurses and two consultant psychiatrists. For this study we interviewed three psychiatric nurses and one psychiatric/psychologist who owns a private practice.

We have provided this information in Results- Study population section.

| **No** | **Position** | **Gender** | **Institution affiliation** |
| --- | --- | --- | --- |
| KI1 | Programme manager | Female | Private not for profit facility |
| KI2 | Psychiatric nurse | Female | Government district hospital |
| KI3 | Psychiatric nurse | Female | Government tertiary hospital |
| KI4 | Director | Male | Ministry of Health |
| KI5 | District Health and Social Service Officer | Male | District hospital |
| KI6 | Private Mental Health Counsellor/Psychiatric consultant | Male | Private practice |
| KI7 | District Health Officer | Female | Ministry of Health |
| KI8 | Psychiatric nurse | Male | Mental health association of Malawi |
| KI9 | Mental hospital director | Female | Government tertiary hospital |

Furthermore, *refer to the systematic review* [*here*](https://www.sciencedirect.com/science/article/pii/S0277953621008558) *that found that a sample size of between 9-17 interviews reached saturation*

4. **Results.** I suggest that Table1 be moved to results section. Otherwise, it still appears to me that the sample size was pre-determined. Even though the study is qualitative, it would improve their manuscript, if the authors added more information on the participants demographics e.g., age, time spent in service, qualifications, levels of health facilities and any other relevant information.

**Response**. Thank you for this comment and suggestion. We included table 1 under study setting and population to provide a description of the people we interviewed. We felt that it made sense that readers have an understanding on the sample before delving into the main results. However, as per your suggestion, we have moved the table to the results section.

We interviewed policy makers and providers of health services- We did not collect information on the age of participants as we did not think it would have an impact on their opinions and perceptions. However, we do agree that including demographic information can enhance the manuscript in certain contexts, but it is not necessary or relevant for the current study's focus.

**Discussion**

Ok

# C. Reviewer #1

Authors have made substantial efforts but the manuscript still needs clarification.

1. Authors entitled the manuscript as « Mental Health is Just an Addendum: Assessing stakeholders’ perceptions on mental health services provision in Malawi prior to and during COVID-19". When we carefully look at the term “prior to” and “during COVID-19”, I do believe that the two periods should be compared in the manuscript.

**Response-** Thank you for this comments and we understand the confusion in the title and have therefore changed it to truly reflect what the study captures. The new title is suggested below

*“Mental Health is Just an Addendum*: assessing stakeholder’s perceptions on Covid-19 and mental health services provision in Malawi”

2. Regarding the sample size, here is again my concern. If there are few mental health services in Malawi, it would have been better to interview all the staff members in each health facilities, if not you have to highlight that as study limitation. If there are only nine people in those health facilities, I totally agree with you. Please clarify which one of the above options you stand on?

**Response**- Thank you for this comment. We appreciate your concerns about the sample size. To clarify, we had a specific target audience of respondents for our study, and we stopped the interviews once we reached the saturation point. We did not want to continue the interviews just to increase the number of respondents beyond the point of saturation. Furthermore, the mental health sector is relatively small, with only three trained psychiatric nurses and two consultant psychiatrists see the paper [here](https://www.ncbi.nlm.nih.gov/pmc/articles/PMC7283112/). Additionally, *refer to the systematic review* [*here*](https://www.sciencedirect.com/science/article/pii/S0277953621008558) *that found that a sample size of between 9-17 interviews reached saturation*. Therefore, we believe our sample is representative of the mental health sector in Malawi.

Thank you for raising this issue, and we have addressed it in the methodology, results and discussion sections.

# D. Reviewer #3

1. The authors have provided a very good background of the topic and justified why the study fits in Malawi.

**Response-** Thank you so much

2. In my view, the authors have addressed most of the concerns raised by the reviewers. The only point no adequate response is made is the general comment made by reviewer 2. The reviewer expressed concern as to why the study has not covered effectiveness of mental health services. No specific response addressed that particular concern either in the manuscript or in the response letter

**Response**- Thank you for your comment. We appreciate your acknowledgment that we addressed most of the concerns raised by the reviewers. We also appreciate the specific concern raised by Reviewer 2 regarding the effectiveness of mental health services, which we did not specifically address in the manuscript or response letter

We apologize for not adequately addressing this concern. Our primary focus was to explore the current state of mental health services in Malawi, including their availability, provision, challenges, and effect of the pandemic. However, we agree that it would have been useful to investigate the effectiveness of mental health services as well, and we will take this suggestion into consideration for future studies

In the conclusion section, we recommended that future research should investigate the effectiveness of mental health service delivery, including assessing the perceptions of beneficiaries. We also suggested that the Malawi Facility Assessment Survey should include mental health-related services to evaluate the quality of care provided and the satisfaction of beneficiaries

Thank you again for bringing this concern to our attention, and we will strive to address it in future research.
