# Supplementary material for: Mental health is just an Addendum: Assessing stakeholder’s perceptions on COVID-19 and mental health services provision in Malawi
Source: PLoS One. 2024 Jun 13;19(6):e0305341. doi: 10.1371/journal.pone.0305341 (PMC11175459; doi:10.1371/journal.pone.0305341)
Supplement: Attachment — Submitted filename: Rebuttal letter.docx [file pone.0305341.s004.docx]

Academic Editor
PLOS ONE

# 21/11/2023

# Dear Editor

# Thank you for inviting us to submit a revised draft of our manuscript entitled, *“Mental Health is Just an Addendum*: assessing stakeholder’s perceptions on COVID-19 and mental health services provision in Malawi” to PLOS ONE. We also appreciate the time and effort you and each of the reviewers have dedicated to providing insightful feedback on ways to strengthen our paper. Thus, it is with great pleasure that we resubmit our article for further consideration. We have incorporated changes that reflect the detailed suggestions you have graciously provided. We also hope that our edits and the responses we provide below satisfactorily address all the issues and concerns you and the reviewers have noted.

To facilitate your review of our revisions, the following is a point-by-point response to the questions and comments

**Reviewer 4**

**Mental Health is Just an Addendum: assessing stakeholder’s perceptions on COVID-19 and mental health services provision in Malawi.**

This is a good study that focused on the integration of mental health services to primary healthcare and in support of the provision of quality healthcare. However, the organization of the manuscript was poorly done and not well written with greater focus on the Abstracts, Methods, Results, and Conclusion sections. These needs to be promptly addressed.


**Abstract**

“…We conducted a formative qualitative assessment using key informant interviews (KIIs) among stakeholders with expert knowledge and involvement of mental health services in Malawi. Data were collected till saturation point. The stakeholders were classified into four policy circles or networks: national government stakeholders, private for-profit stakeholders, public facility hospital managers and district level officers. We conducted semi-structured interviews and, used thematic analysis to explore the data …”
This is the method section of the abstract and inadequate.
Of what need in the categorization on the stakeholders into 4. What does it add to the study? Also, you only interviewed 9 persons.
Look at the example below, which you can improve upon:
“..We conducted a formative qualitative assessment i.e., Key Informant Interview (KIIs) using a semi-structured interview guide among key stakeholders involved in the delivery of mental health services in Malawi. The interviews were audio recorded in English language and was transcribed for thematic analysis by generating codes re-classified into themes, subthemes, and quotes…….

**Response-**Thanks for your suggestion and we have revised the section accordingly

“…..The findings revealed lack of prioritization and inadequate funding for mental health services, which was exacerbated by the COVID-19. Although mental health services are part of the essential health care package and, therefore are supposed to be provided for free in public facilities at all levels, the services are centralized and only functional at a tertiary level of care in public facilities. Lack of medication, shortage of health workers specialized in mental health and poor governance were also identified as challenges that the pandemic exacerbated…..”
This is the result section of the abstract:
Please, this is a result and not a discussion. This is poorly written. What did you find? Looking at your interview guide you can organise your line of though and aligned your result accordingly and in chronological order.

**Response-** The results section has been revised accordingly.

“…..To address these challenges, policymakers in Malawi should prioritise mental health and integrate mental health services into primary healthcare services to expand access. Additionally, policymakers should explore partnerships with the private sector to address shortages of mental health professionals and medication…..”
This is the conclusion and recommendations:
After the findings what was your conclusion with regards to your title and objectives. What you wrote here is farther from it. Kindly look at your study results and give a concise conclusion with appropriate recommendations.
“…Overall, this study highlights the need for policymakers to invest in the mental health sector to improve service quality and efficiency, particularly in light of the COVID-19 pandemic……. This is not a conclusion as most of this was not features in your study. i.e., service quality and efficiency.

**Response-**Thank you so much for this suggestion. The results section has been revised.

**Keywords**: Please use MeSH in selecting your keywords at least 6

**Response-** We have taken time to add more MeSH items

**Introduction**

“…..This paper, therefore, sought to understand from the stakeholders’ perspectives, the state of mental health services provision in a context where resources are limited and how the COVID-19 pandemic affected the provision of the services. We hope that through this study we are engaging with the dialogue on the importance of investing in mental health services in LMICs and strengthen the system for effective provision of the services in the wake of the pandemic and beyond…….” These are the objectives and the justification for the study.

**Response-** Correct.

**Methods**

**Study Design**

“This part informed the development of the semi-stricture interview guide.” Please check the spelling of the semi-structure

**Response-**The sentence has been revised, check the study design section

**Study Settings**

This was grossly inadequate and did not focus on the study setting but rather on data collection method.
I expected the author to give a context of the health service delivery in Malawi with the intersection of mental health service delivery. How is the setup for the health and mental health system in Malawi? How many health facilities, their classifications, and proportion that offer mental health services, what is the spread in the country? What is the administrative structure of Malawi? Total population?
The section gives the audience a general picture of health services and mental health service delivery in Malawi at the time prior to the study.

**Response-**Thank you for this useful suggestion. The study setting has been revised

**Study Population and Sampling**

Bring it here.

**Response-**This has been done

**Data collection tool and method**

Kindly briefly describe some key questions from the interview guide in this section to aid audience’s understanding of your result.

For example: The KII semi-structured interview guide assessed the informant on the awareness of mental health guidelines, policies, and strategies in Malawi, the distribution, availability and access to mental health services, availability of resources for mental health services i.e., human and financial resources, the organization’s role in the provision of mental health services, the impact of COVID-19 on the access and provision of mental health service delivery, and what are the gaps and opportunities experienced in the provision of mental health services resulted from COVID-19 pandemic.

This may give the audience a view of what to expect in your result and it may guide the chronological arrangement of the result. “Transcripts (of what?) were returned to the participants for comments and data validation.” Please, let this be “Transcripts of the audio recordings were returned to the participants for their comments and corroborative data validation”.

**Response-**The section has been revised. And sample questions have been added to the section

**Data analysis**

There is nowhere that you mentioned if the analysis was manual or with a software?

**Response-**We have explicitly mentioned that transcription and analysis was done manually

**Change “Study population” to “Study population and sampling” (move this up to methods).** Start by describing the criteria for the population from which your purposely selected the informants. By the time you describe your study setting, it will justify the population selected and if the number is adequate to generate the result. Though a study says 9 – 17 for saturation may not mean your can be 9 which is a minimum. Remember, you segregated your health organizations to four i.e., Public – Federal, Private non-profit, Private for profit, and Public-District health level or secondary level. How many eligible informants operates at this organizational level or health facilities and how many did you selected from these for interview? Kindly add the sub-section on “Study population and sampling” to the methods section.

**Response-** Thank you for this suggestion. This has been revised

**Results**

This section focused on what was found, and no need to discuss the findings until you reached the discussion section. For example, “Types of services by provider” subsection was not written as a result but as a study setting. The author needs to analyze the informants’ responses as a unit representing Malawi. There is no need differentiating it to the four different health organizations. The respondents’ responses are to be woven into one meaning and supported it with 2 or more quotes. The author can re-organize this section to clearly follow chronological meaning for the audience.
Looking at your questionnaire guide and using the deductive approach, I expected your results with thematic and sub-thematic headings and quotes that address and arranged chronologically as follows:
• awareness of mental health guidelines, policies, and delivery strategies in Malawi
• the distribution, availability, and access to mental health services
• availability of resources for mental health services i.e., human, and financial resources e.t.c.,
• the organization’s role in the provision of mental health services
• the impact of COVID-19 on the access and provision of mental health service delivery during pandemic
• the gaps and opportunities experienced in the provision of mental health services resulted from COVID-19 pandemic.
So, there may be a need for further analysis and bring out findings that address the above with some supportive quotes.
The author can redo Table 2 by looking at the themes and Sub-themes.
Also, there are some conflicts in your result. Primary level is involved in mental health services because they were given tools like guidelines and triaging tools for identification and referral of cases. But you stated the opposite. Involvement and not implementing was different from if you wrote primary level were not involved. This is what I found in your result “so much that health care providers at community and primary levels of care in public facilities lack skills to effectively provide mental health services of any kind….” I think some skills were given but not been implemented. See this “These include (i) the mental health handbook developed in collaboration with the Scotland Malawi health education partnership. Nearly, all facilities at all levels have the book which specifically targets general practitioners who may have had no training in mental health to act as a guideline. Recently, the guide was made available in soft copy and was launched in 2020” and this “At the primary level, mental health illness assessment skills are taken as first aid; that's why there is no specialized care at that level. The expectation for every health worker at this level, whether a nurse, clinical officer, or medical assistant, is the ability to assess and provide treatment to calm a psychotic/aggressive patient before referring them to a higher facility level (KI 2).” So, these inconsistencies are many in the result section.

Also, on results: “Inadequate funding and drug shortages
One of the respondents indicated that another big challenge with providing mental health services is inadequate funding. The WHO estimates that for LMICs to effectively provide primary health care services, they need at least $86, however, the current health expenditure per capita in Malawi is $39.90 (28) which is more than 50% less than what is recommended. This means that even fewer resources are allocated to mental health which is not prioritized but falls under non-communicable diseases (NCDs). This inadequate funding also affects medication availability for mental health-related illnesses, especially in public facilities.” The first statement is a result and good to have a quote. However, the following statements are discussions and not what you found in the analysis. These are kept at the discussion section. This approach is clearer and more appropriate than mixing them up.

**Response-**The whole results section has been revised accordingly.

**Conclusion**
This is not strong and overall conclusion was not well stated by the author. The conclusion should reflect on the title, objectives, and findings from the result to arrive at the overall conclusion from the study found by the authors.
Please, re-write, no conclusion was stated in your conclusion as shown below, but majorly they are recommendations.

Our study engages with the ongoing dialogue on the need for prioritization of mental health services in low- and middle-income countries in the wake of the COVID-19 pandemic. Before any strong recommendations are made, addition studies and analysis are needed in the mental health field to fully unpack the prevalence of mental health disorders in Malawi by sex and age for efficient targeting. We would also need to understand the extend of health system challenges to effectively provide mental health care services for example mapping out available mental health providers, skills, and type of services they can provide. This can be done through the inclusion of mental health related questions in the health facility assessment surveys in Malawi. This will quantify the challenge in effective mental health services delivery and provide a clear understanding of the existing gaps in skills which can then inform appropriate interventions.

Another relevant area of future research would be to understand individual’s perceptions on mental health disorders and how that affect uptake of the services. Studies from Ethiopia (31) and South Africa (32) shows that lack of knowledge and stigma are the main contributing factors that hinder people from seeking mental health services. Finally, given the non-existence of mental health services at lower levels of care, it would also be helpful to understand what interventions are being put in place to promote integration of mental health services at all levels. A report by the WHO and WONCA (20) study shows that integrating mental health services into primary care with effective and acceptable interventions increases access to mental health services.

**Response**-Conclusion has been revised following the recommendation

**These is no information on the limitations to this study. Kindly provide some.**

**Response**-Limitations of the study have been highlighted in the conclusion
