# Supplementary material for: Mental health is just an Addendum: Assessing stakeholder’s perceptions on COVID-19 and mental health services provision in Malawi
Source: PLoS One. 2024 Jun 13;19(6):e0305341. doi: 10.1371/journal.pone.0305341 (PMC11175459; doi:10.1371/journal.pone.0305341)
Supplement: Attachment — Submitted filename: Rebuttal letter.docx [file pone.0305341.s005.docx]

Academic Editor
PLOS ONE

# 21/04/2024

# Dear Editor

# Thank you for inviting us to submit a revised draft of our manuscript entitled, *“Mental Health is Just an Addendum*: assessing stakeholder’s perceptions on COVID-19 and mental health services provision in Malawi” to PLOS ONE. We also appreciate the time and effort you and each of the reviewers have dedicated to providing insightful feedback on ways to strengthen our paper. Thus, it is with great pleasure that we resubmit our article for further consideration. We have incorporated changes that reflect the detailed suggestions you have graciously provided. We also hope that our edits and the responses we provide below satisfactorily address all the issues and concerns you and the reviewers have noted.

To facilitate your review of our revisions, the following is a point-by-point response to the questions and comments.

# **Editors comments**

Thank you for the efforts to address the concerns that have been raised previously. I am satisfied that you have improved your results section. However, your discussion section needs further work. Please expand on why the issues you identified exist e.g. why is there a lack of integration, lack of human resources for mental health etc and how does it compare to other countries in sub-Saharan Africa. What is the evidence from other countries on how these challenges have been overcome and can these strategies be implemented in Malawi? Please move your limitations and recommendations for future research from your conclusion to discussion section. Your conclusion should consist of a maximum of two paragraphs and focus on your key findings and recommendations. At the moment, it reads more like an extension of your discussion.

**Response:** We truly appreciate this important suggestion. Both the discussion and conclusion sections have been revised as suggested.
